# Supplementary material for: Association of Body Mass Index with Risk of Household Catastrophic Health Expenditure in China: A Population-Based Cohort Study
Source: Nutrients. 2022 Sep 27;14(19):4014. doi: 10.3390/nu14194014 (PMC9571178; doi:10.3390/nu14194014)
Supplement: Supplementary file 1 [file nutrients-14-04014-s001.zip › nutrients-1921219-supplementary.pdf]

Table S1. Fully-adjusted Cox proportional hazard model analysis for the association of BMI with risk of CHE.

Characteristics	Number (n = 11185, %)	Events (n = 3275, %)	aHR (95%CI)	p-value
BMI				
Normal	6833 (61.1)	1968 (28.8)	Ref.	—
Lower	831 (7.4)	298 (35.9)	1.15 (1.02–1.31)	0.023
Overweight	2825 (25.3)	822 (29.1)	1.05 (0.97–1.15)	0.211
Obesity	696 (6.2)	187 (26.9)	0.98 (0.84–1.14)	0.746
Gender				
Male	7864 (70.3)	2288 (29.1)	Ref.	—
Female	3321 (29.7)	987 (29.7)	1.05 (0.95–1.15)	0.333
Age group				
16–39	2843 (25.4)	480 (16.9)	Ref.	—
40–49	3452 (30.9)	773 (22.4)	1.23 (1.10–1.38)	<0.001
50–59	2689 (24.0)	965 (35.9)	2.07 (1.85–2.32)	<0.001
≥60	2201 (19.7)	1057 (48.0)	2.85 (2.53–3.22)	<0.001
Marital status				
Married/Partnered	9836 (87.9)	2848 (29.0)	Ref.	—
Other	1349 (12.1)	427 (31.7)	0.86 (0.77–0.97)	0.010
Education				
Illiterate/semiliterate	2732 (24.4)	1028 (37.6)	Ref.	—
Primary school	2649 (23.7)	840 (31.7)	0.98 (0.89–1.08)	0.735
Middle school	3461 (30.9)	894 (25.8)	0.90 (0.82–0.99)	0.038
High school and above	2343 (20.9)	513 (21.9)	0.85 (0.76–0.97)	0.012
Insurance				
None	1394 (12.5)	378 (27.1)	Ref.	—
UEBMI	1354 (12.1)	323 (23.9)	0.94 (0.80–1.10)	0.416
URBMI	757 (6.8)	202 (26.7)	0.95 (0.80–1.13)	0.562
NRCMS	6685 (59.8)	2088 (31.2)	1.00 (0.89–1.12)	0.976
Other	995 (8.9)	284 (28.5)	1.01 (0.86–1.18)	0.933
Current smoking				
No	6053 (54.1)	1774 (29.3)	Ref.	—
Yes	5132 (45.9)	1501 (29.2)	1.00 (0.92–1.09)	0.996
Drinking				
No	8364 (74.8)	2480 (29.7)	Ref.	—
Yes	2821 (25.2)	795 (28.2)	0.93 (0.85–1.01)	0.077
Chronic diseases				
No	9550 (85.4)	2638 (27.6)	Ref.	—
Yes	1635 (14.6)	637 (39.0)	1.22 (1.11–1.33)	<0.001
Self-reported health				
Good	5279 (47.2)	1359 (25.7)	Ref.	—
Medium	4237 (37.9)	1212 (28.6)	0.99 (0.92–1.08)	0.865
Poor	1669 (14.9)	704 (42.2)	1.33 (1.20–1.48)	0.000
Outpatient services				
No	9113 (81.5)	2531 (27.8)	Ref.	—
Yes	2072 (18.5)	744 (35.9)	1.06 (0.97–1.16)	0.225
Inpatient services				
No	10512 (94.0)	3043 (28.9)	Ref.	—
Yes	673 (6.0)	232 (34.5)	1.00 (0.87–1.14)	0.976

Residence				
Urban	5084 (45.5)	1319 (25.9)	Ref.	—
Rural	6101 (54.5)	1956 (32.1)	1.12 (1.04–1.22)	0.005
Family economic level				
Lowest	2941 (26.3)	1058 (36.0)	Ref.	—
Lower	2567 (23.0)	715 (27.9)	0.87 (0.79–0.96)	0.007
Higher	3164 (28.3)	876 (27.7)	0.85 (0.78–0.94)	0.001
Highest	2513 (22.5)	626 (24.9)	0.82 (0.73–0.92)	0.001
Family size				
1–2	2165 (19.4)	885 (40.9)	Ref.	—
3–4	5431 (48.6)	1285 (23.7)	0.73 (0.66–0.80)	<0.001
≥5	3589 (32.1)	1105 (30.8)	0.78 (0.70–0.86)	<0.001
Socioeconomic development level				
Lowest	2358 (21.1)	654 (27.7)	Ref.	—
Lower	3234 (28.9)	981 (30.3)	1.04 (0.94–1.15)	0.458
Higher	2015 (18.0)	637 (31.6)	1.22 (1.09–1.36)	0.001
Highest	3578 (32.0)	1003 (28.0)	1.09 (0.98–1.21)	0.125

Notes: BMI: body mass index; UEBMI: urban employee basic medical insurance; URBMI: urban resident basic medical insurance; NRCMS: new rural cooperative medical scheme; CHE: Catastrophic health expenditure; aHR: adjusted hazard ratio; CI: confident interval; Ref.: Reference.

Table S2. Sensitivity analyses for association of BMI with risk of CHE in total participants and female participants.

BMI groups	Sensitivity analysis 1		Sensitivity analysis 2	
	**aHR (95%CI)**	***p*-value**	**aHR (95%CI)**	***p*-value**
Total				
Normal	Ref.	—	Ref.	—
Underweight	1.16 (1.02–1.31)	0.018	1.15 (1.01–1.30)	0.029
Overweight	1.06 (0.97–1.15)	0.179	1.05 (0.97–1.15)	0.217
Obesity	0.97 (0.84–1.13)	0.731	0.98 (0.84–1.14)	0.798
Female				
Normal	Ref.	—	Ref.	—
Underweight	1.43 (1.17–1.75)	0.001	1.42 (1.16–1.74)	0.001
Overweight	1.28 (1.1–1.48)	0.001	1.25 (1.08–1.46)	0.003
Obesity	1.24 (0.97–1.6)	0.090	1.23 (0.96–1.58)	0.102

Notes: BMI: body mass index; aHR: adjusted hazard ratio; CI: confident interval.
